# The genome sequence of a window fly, *Scenopinus jerei* Pohjoismäki & Haarto, 2021 (Diptera: Scenopinidae)

**DOI:** 10.12688/wellcomeopenres.26115.1

**Published:** 2026-03-18

**Authors:** Jaakko Pohjoismäki, Wellcome Sanger Institute Tree of Life Management, Samples and Laboratory team, Wellcome Sanger Institute Scientific Operations: Sequencing Operations, Wellcome Sanger Institute Tree of Life Core Informatics team, Tree of Life Core Informatics collective, Mark L. Blaxter

**Affiliations:** 1 Department of Environmental and Biological Sciences, University of Eastern Finland, Joensuu, Finland; 2 Tree of Life Programme, Wellcome Sanger Institute, Hinxton, England, UK

**Keywords:** Scenopinus jerei; window fly; genome sequence; chromosomal; Diptera

## Abstract

We present a genome assembly from an individual male *Scenopinus jerei* (window fly; Arthropoda; Insecta; Diptera; Scenopinidae). The assembly contains two haplotypes with total lengths of 345.25 megabases and 232.44 megabases. Most of haplotype 1 (94.85%) is scaffolded into 5 chromosomal pseudomolecules, including the X and Y sex chromosomes. Haplotype 2 was assembled to scaffold level. The mitochondrial genome has also been assembled, with a length of 16.52 kilobases. This assembly was generated as part of the Darwin Tree of Life project, which produces reference genomes for eukaryotic species found in Britain and Ireland.

## Species taxonomy

Eukaryota; Opisthokonta; Metazoa; Eumetazoa; Bilateria; Protostomia; Ecdysozoa; Panarthropoda; Arthropoda; Mandibulata; Pancrustacea; Hexapoda; Insecta; Dicondylia; Pterygota; Neoptera; Endopterygota; Diptera; Brachycera; Muscomorpha; Asiloidea; Scenopinidae; *Scenopinus*; *Scenopinus jerei* Pohjoismäki & Haarto, 2021 (NCBI:txid2969622).

## Background

*Scenopinus jerei* Pohjoismäki and Haarto, 2021 is a member of the family Scenopinidae, commonly known as window flies. Scenopinidae are a small, cosmopolitan family of primitive flies within the therevoid clade of Asiloidea, with over 420 described species in 25 genera ( Winterton & Gaimari, 2017; Winterton & Ware, 2015). In total, 18 species of Scenopinidae are known from Europe and all but one ( *Caenoneura nigra* Kelsey, 1969) belong to the genus *Scenopinus* Latreille, 1802 ( Pohjoismäki & Haarto, 2021). Three species occur in Finland: the cosmopolitan *S. fenestralis* (Linnaeus, 1758), *S. niger* (De Geer, 1776), and *S. jerei* ( Kahanpää *et al.*, 2014; Pohjoismäki *et al.*, 2023; Pohjoismäki & Haarto, 2021). Only *S. fenestralis* and *S. niger* have been reported from the UK ( Stubbs & Drake, 2014).

**Figure 1.**
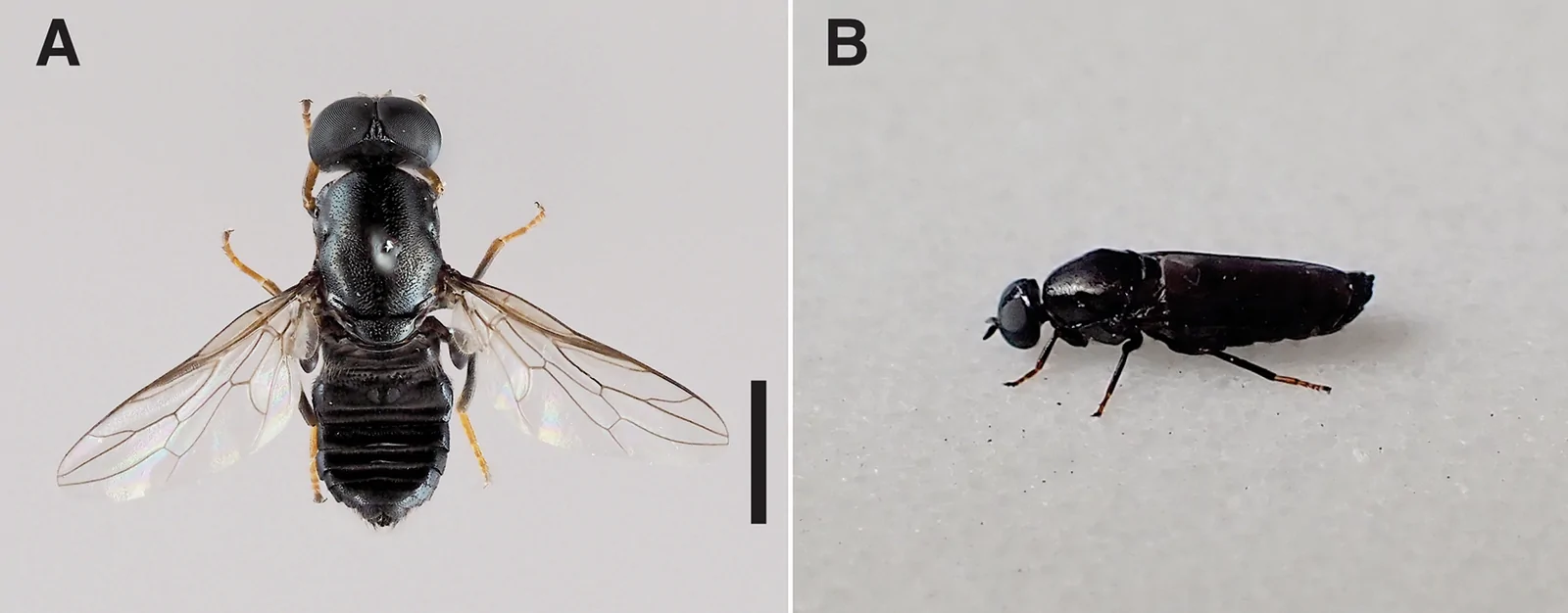
A. Male *Scenopinus jerei* serving as a proxy voucher specimen ( http://tun.fi/JX.1409450), collected on 2022-06-25 from the same locality as idSceJere3. Ilomantsi, Kelovaara, Finland (N63.062, E30.610), which is also the paratype locality of the species ( Pohjoismäki and Haarto, 2021). Photo: Jiri Vihavainen. Scale bar 1 mm. B. Female specimen (idSceJere2) from same the locality and collection event, used for RNA sequencing. Photo: Jaakko Pohjoismäki.

Adults of *Scenopinus jerei* are 4–6 mm long, with dark brown to black colouration, clear wings, and distinctly bicoloured legs: coxae and femora are dark, whereas tibiae are paler and often orange ( Figure 1). For a more detailed description and a key to the European species, see Pohjoismäki and Haarto (2021). Adults are typically encountered from mid- to end June.

The species inhabits boreal forest environments and has been recorded from Finland, Sweden, and Russian Karelia, with a likely wider distribution across the boreal forest zone of the Palaearctic. In Finland it is widespread in southern and central parts of the country, but rarely observed. *Scenopinus jerei* larvae develop in the nests of cavity-breeding birds such as tits and owls, where they most likely prey on other invertebrates including larvae of fleas and tineid moths. Like other window flies, it may occasionally be found indoors, particularly in unheated buildings. Due to its elusive biology and generally limited taxonomic information about Scenopinidae, the species was not recognised until recently and was described as new to science in 2021 ( Pohjoismäki & Haarto, 2021). As the species has a broad distribution range and an abundance of suitable habitats, *S. jerei* is currently assessed as Least Concern (LC) in Finland.

The reference genome assembly of *Scenopinus jerei* provides the first genomic resource for Scenopinidae. It contributes to the growing representation of the therevoid clade in genomic databases and supports studies on the evolution of predatory and commensal life histories in asiloid flies. Furthermore, it offers a foundation for future research on biodiversity genomics in boreal forest ecosystems and bird nest invertebrate communities.

## Methods

### Sample acquisition

The specimen used for genome sequencing was an adult male *Scenopinus jerei* (specimen ID SAN20001196, ToLID idSceJere3), collected from Kelovaara, Ilomantsi, North Karelia, Finland (latitude 63.0635, longitude 30.6071) on 2022-06-25. Another specimen was used for RNA sequencing (specimen ID SAN20001197, ToLID idSceJere2). It was collected from Kelovaara, Ilomantsi, North Karelia, Finland (latitude 63.0635, longitude 30.6071) on 2022-06-25. Both specimens were collected and identified by Jaakko Pohjoismaki.

### Nucleic acid extraction

Protocols for high molecular weight (HMW) DNA extraction developed at the Wellcome Sanger Institute (WSI) Tree of Life Core Laboratory are available on protocols.io ( Howard *et al.*, 2025). The idSceJere3 sample was weighed and triaged to determine the appropriate extraction protocol. Tissue from the whole organism was homogenised by powermashing using a PowerMasher II tissue disruptor. HMW DNA was extracted using the Automated MagAttract v2 protocol. We used centrifuge-mediated fragmentation to produce DNA fragments in the 8–10 kb range, following the Covaris g-TUBE protocol for ultra-low input (ULI). Sheared DNA was purified by automated SPRI (solid-phase reversible immobilisation). The concentration of the sheared and purified DNA was assessed using a Nanodrop spectrophotometer and Qubit Fluorometer using the Qubit dsDNA High Sensitivity Assay kit. Fragment size distribution was evaluated by running the sample on the FemtoPulse system. For this sample, the final post-shearing DNA had a Qubit concentration of 1.67 ng/μL and a yield of 217.10 ng.

RNA was extracted from whole organism tissue of idSceJere2 in the Tree of Life Laboratory at the WSI using the RNA Extraction: Automated MagMax™ *mir*Vana protocol. The RNA concentration was assessed using a Nanodrop spectrophotometer and a Qubit Fluorometer using the Qubit RNA Broad-Range Assay kit. Analysis of the integrity of the RNA was done using the Agilent RNA 6000 Pico Kit and Eukaryotic Total RNA assay.

### PacBio HiFi library preparation and sequencing

Library preparation and sequencing were performed at the WSI Scientific Operations core. Prior to library preparation, the DNA was fragmented to ~10 kb. Ultra-low-input (ULI) libraries were prepared using the PacBio SMRTbell ^®^ Express Template Prep Kit 2.0 and gDNA Sample Amplification Kit. Samples were normalised to 20 ng DNA. Single-strand overhang removal, DNA damage repair, and end-repair/A-tailing were performed according to the manufacturer’s instructions, followed by adapter ligation. A 0.85× pre-PCR clean-up was carried out with Promega ProNex beads.

The DNA was evenly divided into two aliquots for dual PCR (reactions A and B), both following the manufacturer’s protocol. A 0.85× post-PCR clean-up was performed with ProNex beads. DNA concentration was measured using a Qubit Fluorometer v4.0 (Thermo Fisher Scientific) with the Qubit HS Assay Kit, and fragment size was assessed on an Agilent Femto Pulse Automated Pulsed Field CE Instrument (Agilent Technologies) using the gDNA 55 kb BAC analysis kit. PCR reactions A and B were then pooled, ensuring a total mass of ≥500 ng in 47.4 μl.

The pooled sample underwent another round of DNA damage repair, end-repair/A-tailing, and hairpin adapter ligation. A 1× clean-up was performed with ProNex beads, followed by DNA quantification using the Qubit and fragment size analysis using the Agilent Femto Pulse. Size selection was performed on the Sage Sciences PippinHT system, with target fragment size determined by Femto Pulse analysis (typically 4–9 kb). Size-selected libraries were cleaned with 1.0× ProNex beads and normalised to 2 nM before sequencing.

The sample was sequenced on a Revio instrument (Pacific Biosciences). The prepared library was normalised to 2 nM, and 15 μL was used for making complexes. Primers were annealed and polymerases bound to generate circularised complexes, following the manufacturer’s instructions. Complexes were purified using 1.2X SMRTbell beads, then diluted to the Revio loading concentration (200–300 pM) and spiked with a Revio sequencing internal control. The sample was sequenced on a Revio 25M SMRT cell. The SMRT Link software (Pacific Biosciences), a web-based workflow manager, was used to configure and monitor the run and to carry out primary and secondary data analysis.

### Hi-C

***Sample preparation and crosslinking***

The Hi-C sample was prepared from 20–50 mg of frozen tissue from the idSceJere3 sample using the Arima-HiC v2 kit (Arima Genomics). Following the manufacturer’s instructions, tissue was fixed and DNA crosslinked using TC buffer to a final formaldehyde concentration of 2%. The tissue was homogenised using the Diagnocine Power Masher-II. Crosslinked DNA was digested with a restriction enzyme master mix, biotinylated, and ligated. Clean-up was performed with SPRISelect beads before library preparation. DNA concentration was measured with the Qubit Fluorometer (Thermo Fisher Scientific) and Qubit HS Assay Kit. The biotinylation percentage was estimated using the Arima-HiC v2 QC beads.

***Hi-C library preparation and sequencing***

Biotinylated DNA constructs were fragmented using a Covaris E220 sonicator and size selected to 400–600 bp using SPRISelect beads. DNA was enriched with Arima-HiC v2 kit Enrichment beads. End repair, A-tailing, and adapter ligation were carried out with the NEBNext Ultra II DNA Library Prep Kit (New England Biolabs), following a modified protocol where library preparation occurs while DNA remains bound to the Enrichment beads. Library amplification was performed using KAPA HiFi HotStart mix and a custom Unique Dual Index (UDI) barcode set (Integrated DNA Technologies). Depending on sample concentration and biotinylation percentage determined at the crosslinking stage, libraries were amplified with 10–16 PCR cycles. Post-PCR clean-up was performed with SPRISelect beads. Libraries were quantified using the AccuClear Ultra High Sensitivity dsDNA Standards Assay Kit (Biotium) and a FLUOstar Omega plate reader (BMG Labtech).

Prior to sequencing, libraries were normalised to 10 ng/μL. Normalised libraries were quantified again to create equimolar and/or weighted 2.8 nM pools. Pool concentrations were checked using the Agilent 4200 TapeStation (Agilent) with High Sensitivity D500 reagents before sequencing. Sequencing was performed using paired-end 150 bp reads on the Illumina NovaSeq 6000.

### RNA library preparation and sequencing

Libraries were prepared using the NEBNext ^®^ Ultra™ II Directional RNA Library Prep Kit for Illumina (New England Biolabs), following the manufacturer’s instructions. Poly(A) mRNA in the total RNA solution was isolated using oligo (dT) beads, converted to cDNA, and uniquely indexed; 14 PCR cycles were performed. Libraries were size-selected to produce fragments between 100–300 bp. Libraries were quantified, normalised, pooled to a final concentration of 2.8 nM, and diluted to 150 pM for loading. Sequencing was carried out on the Illumina NovaSeq 6000, generating paired-end reads.

### Genome assembly

Prior to assembly of the PacBio HiFi reads, a database of *k*-mer counts ( *k* = 31) was generated from the filtered reads using FastK. GenomeScope2 ( Ranallo-Benavidez *et al.*, 2020) was used to analyse the *k*-mer frequency distributions, providing estimates of genome size, heterozygosity, and repeat content.

The HiFi reads were assembled using Hifiasm in Hi-C phasing mode ( Cheng *et al.*, 2021, 2022), producing two haplotypes. Hi-C reads ( Rao *et al.*, 2014) were mapped to the primary contigs using bwa-mem2 ( Vasimuddin *et al.*, 2019). Contigs were further scaffolded with Hi-C data in YaHS ( Zhou *et al.*, 2023), using the --break option for handling potential misassemblies. The scaffolded assemblies were evaluated using Gfastats ( Formenti *et al.*, 2022), BUSCO ( Manni *et al.*, 2021) and MERQURY.FK ( Rhie *et al.*, 2020).

The mitochondrial genome was assembled using MitoHiFi ( Uliano-Silva *et al.*, 2023).

### Assembly curation

The assembly was decontaminated using the Assembly Screen for Cobionts and Contaminants ( ASCC) pipeline. TreeVal was used to generate the flat files and maps for use in curation. Manual curation was conducted primarily in PretextView and HiGlass ( Kerpedjiev *et al.*, 2018). Scaffolds were visually inspected and corrected as described by Howe *et al*. (2021). Manual corrections included 58 breaks and 261 joins. This reduced the scaffold count by 21.6%, increased the scaffold N50 by 7.7%, and increased the total assembly length by 4.4%. The curation process is described at https://gitlab.com/wtsi-grit/rapid-curation . PretextSnapshot was used to generate a Hi-C contact map of the final assembly.

### Assembly quality assessment

The Merqury.FK tool ( Rhie *et al.*, 2020) was run in a Singularity container ( Kurtzer *et al.*, 2017) to evaluate *k*-mer completeness and assembly quality for both haplotypes using the *k*-mer databases ( *k* = 31) computed prior to genome assembly. The analysis outputs included assembly QV scores and completeness statistics.

The genome was analysed using the BlobToolKit pipeline, a Nextflow implementation of the earlier Snakemake version ( Challis *et al.*, 2020). The pipeline aligns PacBio reads using minimap2 ( Li, 2018) and SAMtools ( Danecek *et al.*, 2021) to generate coverage tracks. It runs BUSCO ( Manni *et al.*, 2021) using lineages identified from the NCBI Taxonomy ( Schoch *et al.*, 2020). For the three domain-level lineages, BUSCO genes are aligned to the UniProt Reference Proteomes database ( Bateman *et al.*, 2023) using DIAMOND blastp ( Buchfink *et al.*, 2021). The genome is divided into chunks based on the density of BUSCO genes from the closest taxonomic lineage, and each chunk is aligned to the UniProt Reference Proteomes database with DIAMOND blastx. Sequences without hits are chunked using seqtk and aligned to the NT database with blastn ( Altschul *et al.*, 1990). The BlobToolKit suite consolidates all outputs into a blobdir for visualisation. The BlobToolKit pipeline was developed using nf-core tooling ( Ewels *et al.*, 2020) and MultiQC ( Ewels *et al.*, 2016), with containerisation through Docker ( Merkel, 2014) and Singularity ( Kurtzer *et al.*, 2017).

## Genome sequence report

### Sequence data

**Figure 2.**
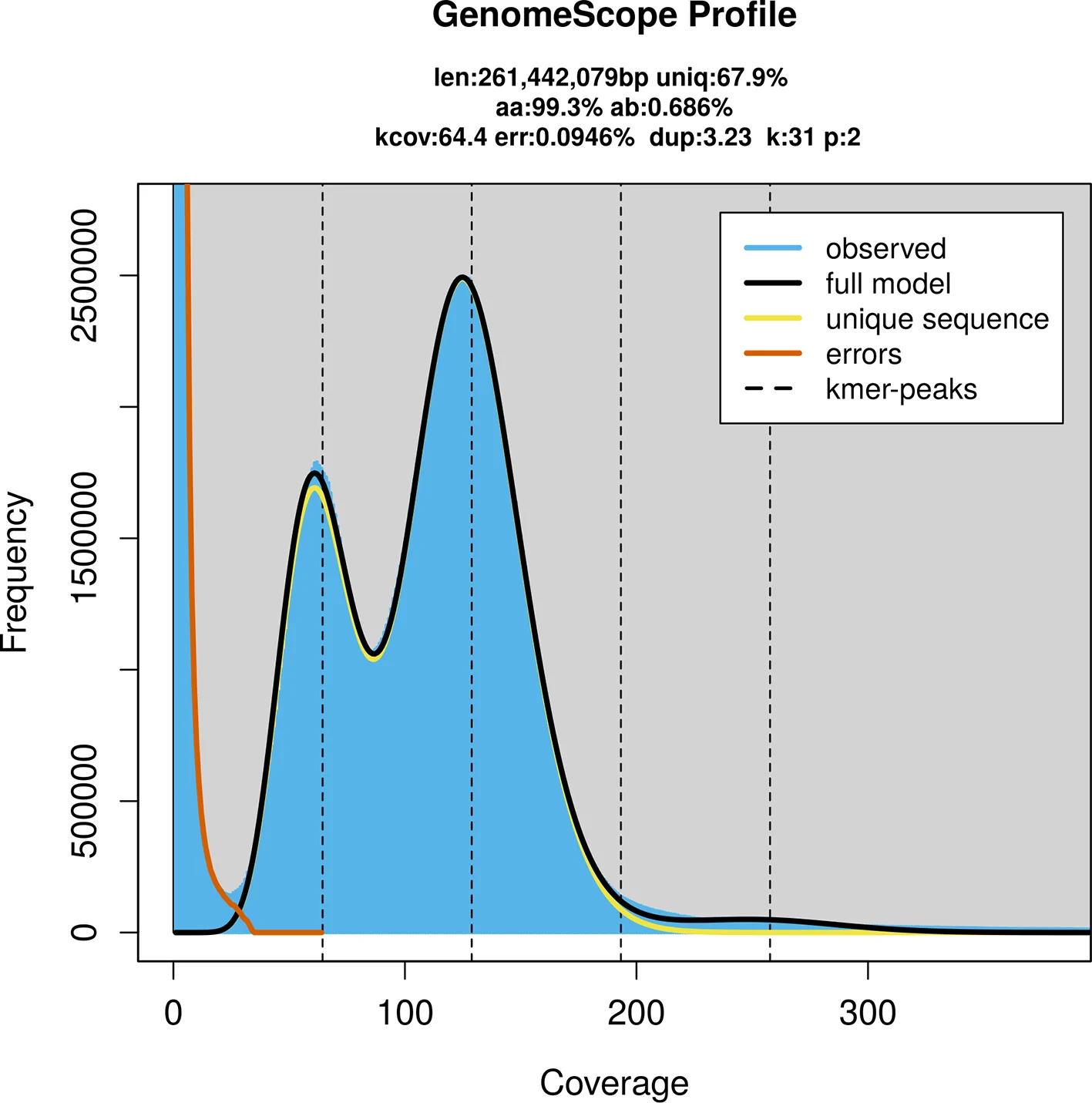
Frequency distribution of *k*-mers generated using GenomeScope2. The plot shows observed and modelled *k*-mer spectra, providing estimates of genome size, heterozygosity, and repeat content based on unassembled sequencing reads.

PacBio sequencing of the *Scenopinus jerei* specimen generated 35.72 Gb (gigabases) from 4.21 million reads, which were used to assemble the genome. GenomeScope2.0 analysis estimated the haploid genome size at 261.67 Mb, with a heterozygosity of 0.69% and repeat content of 32.08% ( Figure 2). These estimates guided expectations for the assembly. Based on the estimated genome size, the sequencing data provided approximately 129× coverage. Hi-C sequencing produced 258.39 Gb from 1 711.19 million reads, which were used to scaffold the assembly. RNA sequencing data were also generated and are available in public sequence repositories. Table 1 summarises the specimen and sequencing details.

**Table 1.** Specimen and sequencing data for BioProject PRJEB68024.

Platform	PacBio HiFi	Hi-C	RNA-seq
**ToLID**	idSceJere3	idSceJere3	idSceJere2
**Specimen ID**	SAN20001196	SAN20001196	SAN20001197
**BioSample (source individual)**	SAMEA112142239	SAMEA112142239	SAMEA112142238
**BioSample (tissue)**	SAMEA112142241	SAMEA112142241	SAMEA112142240
**Tissue**	whole organism	whole organism	whole organism
**Instrument**	Revio	Illumina NovaSeq 6000	Illumina NovaSeq 6000
**Run accessions**	ERR12205288	ERR12245618; ERR12245617	ERR12245619
**Read count total**	4.21 million	1 711.19 million	71.47 million
**Base count total**	35.72 Gb	258.39 Gb	10.79 Gb

### Assembly statistics

The genome was assembled into two haplotypes using Hi-C phasing. Haplotype 1 was curated to chromosome level, while haplotype 2 was assembled to scaffold level. The final assembly has a total length of 345.25 Mb in 523 scaffolds, with 374 gaps, and a scaffold N50 of 75.08 Mb ( Table 2).

**Table 2.** Genome assembly statistics.

**Assembly name**	idSceJere3.hap1.1	idSceJere3.hap2.1
**Assembly accession**	GCA_965641765.1	GCA_965641775.1
**Assembly level**	chromosome	scaffold
**Span (Mb)**	345.25	232.44
**Number of chromosomes**	5	-
**Number of contigs**	897	446
**Contig N50**	1.83 Mb	3.21 Mb
**Number of scaffolds**	523	281
**Scaffold N50**	75.08 Mb	73.56 Mb
**Longest scaffold length (Mb)**	81.54	-
**Sex chromosomes**	X and Y	-
**Organelles**	Mitochondrion: 16.52 kb	-

**Figure 3.**
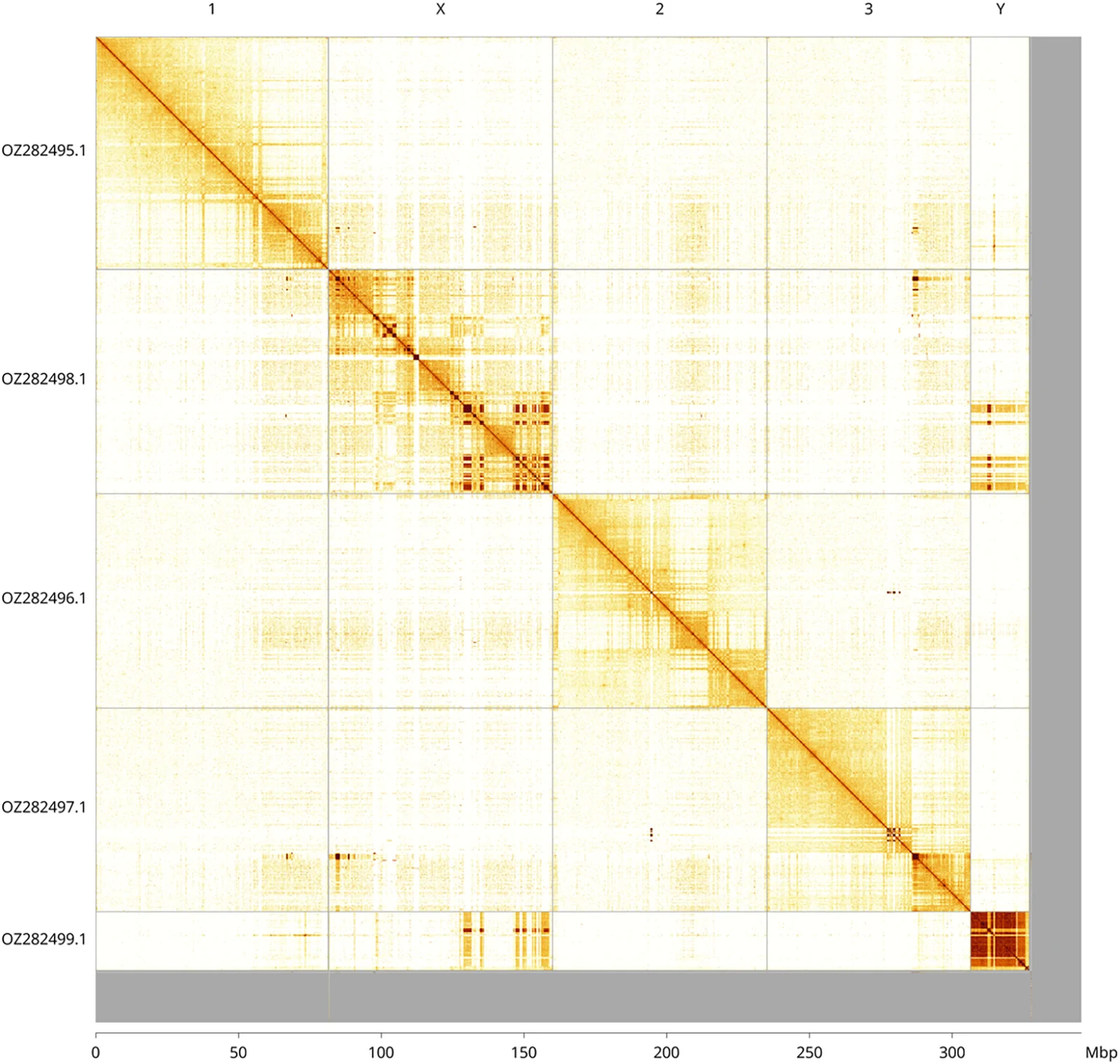
Hi-C contact map of the *Scenopinus jerei* genome assembly. Assembled chromosomes are shown in order of size and labelled along the axes, with a megabase scale shown below. The plot was generated using PretextSnapshot.

Most of the haplotype 1 assembly sequence (94.85%) was assigned to 5 chromosomal-level scaffolds, representing 3 autosomes and the X and Y sex chromosomes. These chromosome-level scaffolds, confirmed by Hi-C data, are named according to size ( Figure 3; Table 3). The exact order and orientation of the contigs on chromosome Y and X is unknown and is based mostly on Hi-C signal. The sex chromosomes were identified by read coverage. The assignement of X and Y was based on heterochromatic region size and BUSCO (diptera_odb12) gene count.

**Table 3.** Chromosomal pseudomolecules in the haplotype 1 genome assembly of *Scenopinus jerei* idSceJere3.

INSDC accession	Molecule	Length (Mb)	GC%
OZ282495.1	1	81.61	33
OZ282496.1	2	75.08	33
OZ282497.1	3	71.30	33
OZ282498.1	X	78.49	36
OZ282499.1	Y	21	39.50

The mitochondrial genome was also assembled (length 16.52 kb, OZ282500.1). This sequence is included as a contig in the multifasta file of the genome submission and as a standalone record.

### Assembly quality metrics

**Figure 4.**
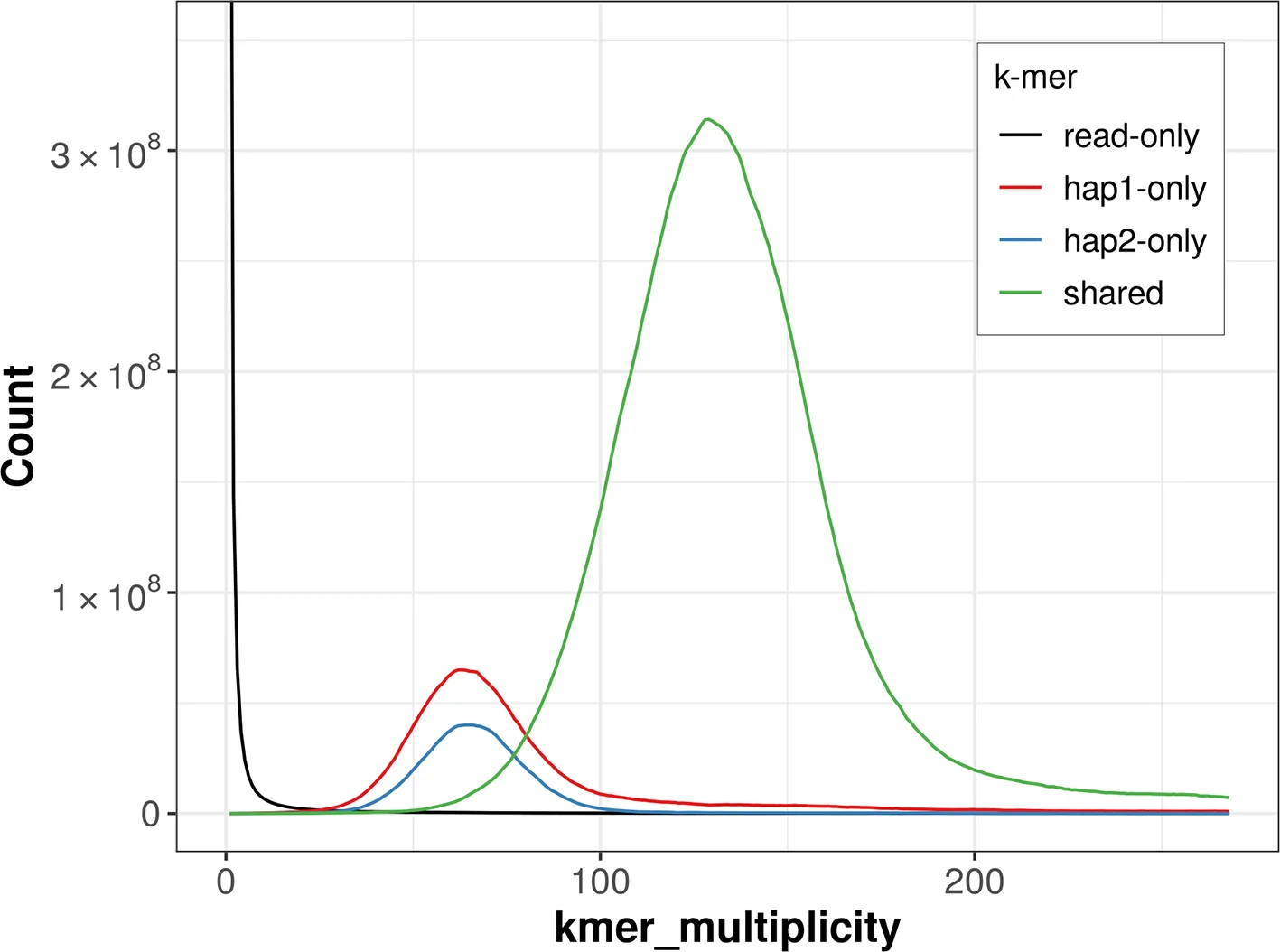
Evaluation of *k*-mer completeness using MerquryFK. This plot illustrates the recovery of *k*-mers from the original read data in the final assemblies. The horizontal axis represents *k*-mer multiplicity, and the vertical axis shows the number of *k*-mers. The black curve represents *k*-mers that appear in the reads but are not assembled. The green curve corresponds to *k*-mers shared by both haplotypes, and the red and blue curves show *k*-mers found only in one of the haplotypes.

For haplotype 1, the estimated QV is 61.2, and for haplotype 2, 62.7. When the two haplotypes are combined, the assembly achieves an estimated QV of 61.7. The *k*-mer completeness is 90.09% for haplotype 1, 80.79% for haplotype 2, and 99.53% for the combined haplotypes ( Figure 4).

**Figure 5.**
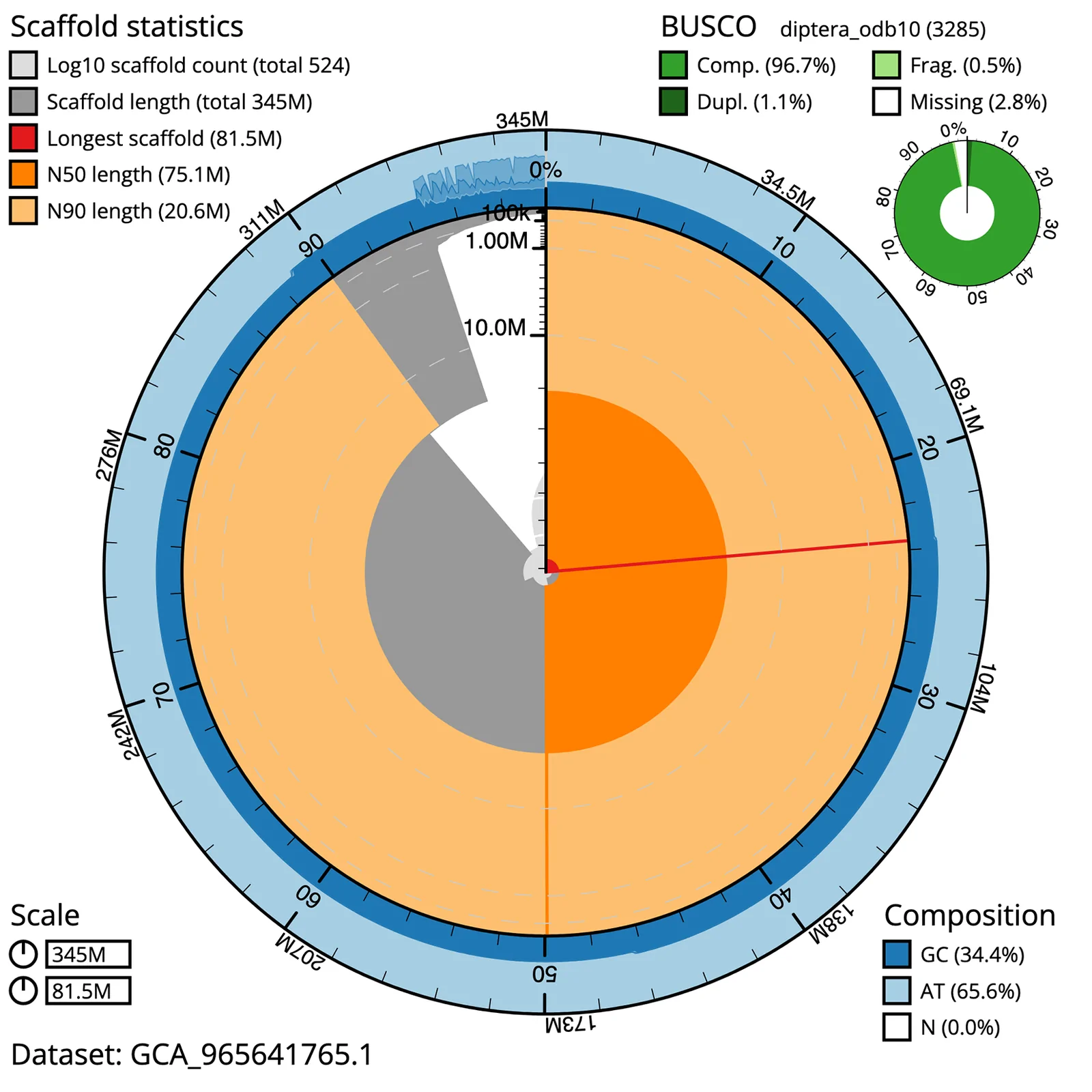
Assembly metrics for idSceJere3.hap1.1. The BlobToolKit snail plot provides an overview of assembly metrics and BUSCO gene completeness. The circumference represents the length of the whole genome sequence, and the main plot is divided into 1 000 bins around the circumference. The outermost blue tracks display the distribution of GC, AT, and N percentages across the bins. Scaffolds are arranged clockwise from longest to shortest and are depicted in dark grey. The longest scaffold is indicated by the red arc, and the deeper orange and pale orange arcs represent the N50 and N90 lengths. A light grey spiral at the centre shows the cumulative scaffold count on a logarithmic scale. A summary of complete, fragmented, duplicated, and missing BUSCO genes in the set is presented at the top right. An interactive version of this figure can be accessed on the BlobToolKit viewer.

**Figure 6.**
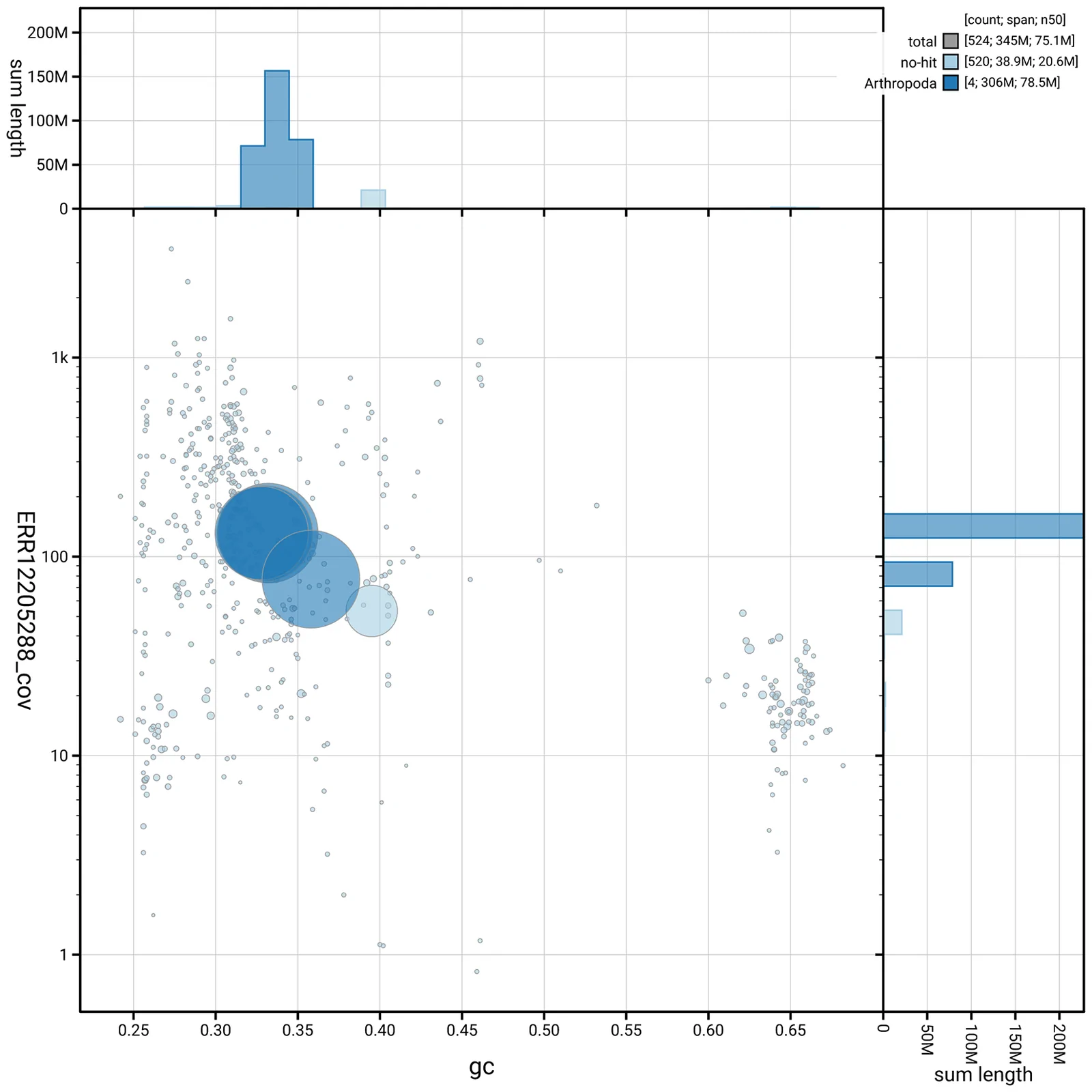
BlobToolKit blob plot for idSceJere3.hap1.1. The plot shows base coverage (vertical axis) and GC content (horizontal axis). The circles represent scaffolds, with the size proportional to scaffold length and the colour representing phylum membership. The histograms along the axes display the total length of sequences distributed across different levels of coverage and GC content. An interactive version of this figure is available on the BlobToolKit viewer.

BUSCO analysis using the endopterygota_odb10 reference set ( *n* = 2 124) identified 98.4% of the expected gene set (single = 97.4%, duplicated = 1.0%) in haplotype 1. For haplotype 2, BUSCO v.5.8.3 analysis identified 96.8% of the expected gene set (single = 96.0%, duplicated = 0.8%). The snail plot in Figure 5 summarises the scaffold length distribution and other assembly statistics for haplotype 1. The blob plot in Figure 6 shows the distribution of scaffolds by GC proportion and coverage for haplotype 1.

Table 4 lists the assembly metric benchmarks adapted from Rhie *et al.* (2021) and the Earth BioGenome Project Report on Assembly Standards September 2024. The EBP metric, calculated for the haplotype 1, is **6.C.Q61**, meeting the recommended reference standard.

**Table 4.** Earth Biogenome Project summary metrics for the *Scenopinus jerei* assembly.

Measure	Value	Benchmark
EBP summary (haplotype 1)	6.C.Q61	6.C.Q40
Contig N50 length	1.83 Mb	≥ 1 Mb
Scaffold N50 length	75.08 Mb	= chromosome N50
Consensus quality (QV)	Haplotype 1: 61.2; haplotype 2: 62.7; combined: 61.7	≥ 40
*k*-mer completeness	Haplotype 1: 90.09%; haplotype 2: 80.79%; combined: 99.53%	≥ 95%
BUSCO	C:98.4% [S:97.4%; D:1.0%]; F:0.4%; M:1.2%; n:2 124	S > 90%; D < 5%
Percentage of assembly assigned to chromosomes	94.85%	≥ 90%

**Notes:** EBP summary uses log10(Contig N50); chromosome-level (C) or log10(Scaffold N50); Q (Merqury QV). BUSCO: C=complete; S=single-copy; D=duplicated; F=fragmented; M=missing; n=orthologues using the diptera_odb10 reference set.

**Table 5.** Software versions and sources.

Software	Version	Source
BEDTools	2.30.0	https://github.com/arq5x/bedtools2
BLAST	2.14.0	ftp://ftp.ncbi.nlm.nih.gov/blast/executables/blast+/
BlobToolKit	4.4.6	https://github.com/blobtoolkit/blobtoolkit
BUSCO	5.8.3	https://gitlab.com/ezlab/busco
bwa-mem2	2.2.1	https://github.com/bwa-mem2/bwa-mem2
Cooler	0.8.11	https://github.com/open2c/cooler
DIAMOND	2.1.8	https://github.com/bbuchfink/diamond
fasta_windows	0.2.4	https://github.com/tolkit/fasta_windows
FastK	1.1	https://github.com/thegenemyers/FASTK
GenomeScope2.0	2.0.1	https://github.com/tbenavi1/genomescope2.0
Gfastats	1.3.6	https://github.com/vgl-hub/gfastats
Hifiasm	0.19.8-r603	https://github.com/chhylp123/hifiasm
HiGlass	1.13.4	https://github.com/higlass/higlass
MerquryFK	1.1.2	https://github.com/thegenemyers/MERQURY.FK
Minimap2	2.28-r1209	https://github.com/lh3/minimap2
MitoHiFi	3	https://github.com/marcelauliano/MitoHiFi
MultiQC	1.14; 1.17 and 1.18	https://github.com/MultiQC/MultiQC
Nextflow	24.10.4	https://github.com/nextflow-io/nextflow
PretextSnapshot	0.0.5	https://github.com/sanger-tol/PretextSnapshot
PretextView	1.0.3	https://github.com/sanger-tol/PretextView
samtools	1.21	https://github.com/samtools/samtools
sanger-tol/ascc	0.1.0	https://github.com/sanger-tol/ascc
sanger-tol/blobtoolkit	v0.8.0	https://github.com/sanger-tol/blobtoolkit
sanger-tol/curationpretext	1.4.2	https://github.com/sanger-tol/curationpretext
Seqtk	1.3	https://github.com/lh3/seqtk
Singularity	3.9.0	https://github.com/sylabs/singularity
TreeVal	1.4.0	https://github.com/sanger-tol/treeval
YaHS	1.2a.2	https://github.com/c-zhou/yahs

## Author information

Contributors are listed at the following links:

• Members of the Wellcome Sanger Institute Tree of Life Management, Samples and Laboratory team
• Members of Wellcome Sanger Institute Scientific Operations – Sequencing Operations
• Members of the Wellcome Sanger Institute Tree of Life Core Informatics team
• Members of the Tree of Life Core Informatics collective

## Wellcome Sanger Institute – Legal and Governance

The materials that have contributed to this genome note have been supplied by a Tree of Life collaborator. The Wellcome Sanger Institute employs a process whereby due diligence is carried out proportionate to the nature of the materials themselves, and the circumstances under which they have been/are to be collected and provided for use. The purpose of this is to address and mitigate any potential legal and/or ethical implications of receipt and use of the materials as part of the research project, and to ensure that in doing so, we align with best practice wherever possible. The overarching areas of consideration are:

• Ethical review of provenance and sourcing of the material.
• Legality of collection, transfer and use (national and international).

Each transfer of samples is undertaken according to a Research Collaboration Agreement or Material Transfer Agreement entered into by the Tree of Life collaborator, Genome Research Limited (operating as the Wellcome Sanger Institute), and in some circumstances, other Tree of Life collaborators.

## Data Availability

European Nucleotide Archive: Scenopinus jerei. Accession number PRJEB68024 ( https://identifiers.org/ena.embl/PRJEB68024). The genome sequence is released openly for reuse. The *Scenopinus jerei* genome sequencing initiative is part of the Sanger Institute Tree of Life Programme (PRJEB43745). All raw sequence data and the assembly have been deposited in INSDC databases. The genome will be annotated using available RNA-Seq data and presented through the Ensembl pipeline at the European Bioinformatics Institute. Raw data and assembly accession identifiers are reported in Tables 1 and 2. Production code used in genome assembly at the WSI Tree of Life is available at https://github.com/sanger-tol . Table 5 lists software versions used in this study.
