# Supplementary material for: The genome sequence of a window fly, Scenopinus jerei Pohjoismäki & Haarto, 2021 (Diptera: Scenopinidae)
Source: Wellcome Open Res. 2026 Mar 18;11:167. [Version 1] doi: 10.12688/wellcomeopenres.26115.1 (PMC13054250; doi:10.12688/wellcomeopenres.26115.1)
Supplement: Peer review history

# Reviewer report: Reviewer response for version 1

**DOI:** 10.21956/wellcomeopenres.28758.r151704

**Published:** 2026-09-01

**Authors:** Rajib Deb

**Affiliations:** 1 ICAR-National Research Centre, Guwahat, Assam, India

Minor concerns

 1. Please clarify why the two haplotypes differ substantially in assembly size (345.25 Mb and 232.44 Mb) -abstract

 2. Kindly provide any additional information on morphological/molecular identification of sequenced male specimen

 3. Provide details supporting the X and Y chromosomes assignment

 4. The final DNA concentration was only 1.67 ng/µL, with a total yield of 217.10 ng. Please clarify whether this low input affected library preparation or sequencing performance

Are sufficient details of methods and materials provided to allow replication by others?

Yes

Is the rationale for creating the dataset(s) clearly described?

Yes

Are the datasets clearly presented in a useable and accessible format?

Yes

Are the protocols appropriate and is the work technically sound?

Yes

Reviewer Expertise:

Genomics

I confirm that I have read this submission and believe that I have an appropriate level of expertise to confirm that it is of an acceptable scientific standard, however I have significant reservations, as outlined above.

# Reviewer report: Reviewer response for version 1

**DOI:** 10.21956/wellcomeopenres.28758.r151707

**Published:** 2026-04-10

**Authors:** Zheng Li, Maksudul Islam

**Affiliations:** 1 Biology, Miami University Department of Biology (Ringgold ID: 124576), Oxford, Ohio, USA

The author presents the genome assembly with two haplotypes of Scenopinus jerei (window fly). Haplotype 1 spans 345.25 Mb, with most of the sequence (94.85%) assembled into 5 chromosomal-level scaffolds. Haplotype 2 is assembled at the scaffold level with 232.44 Mb. The authors provide background on the species taxonomy, anatomy, geographic distribution, and habitat. Each method section is detailed with adequate information to increase reproducibility. The assembly exhibits high completeness, benchmarked with different quality metrics including a BUSCO score of 98.4%. This genome will serve as a valuable resource for studying Scenopinidae comparative genomics, as it is the first genomic resource and will provide important insights into genome evolution and biodiversity. Here are some minor issues that should be addressed:

- There is a spelling error that should be corrected: "The assignement of X and Y was based on heterochromatic region size and BUSCO (diptera_odb12) gene count."  "assignement" should be corrected to "assignment".
- Table 2 reports the longest scaffold for haplotype 1 as 81.54 Mb, whereas Table 3 reports the longest chromosome (OZ282495.1) as 81.61 Mb. This discrepancy should be clarified for consistency.

- The BUSCO analysis is unclear. The snail plot (Figure 5) presents BUSCO results using the diptera_odb10 reference set, whereas the text reports completeness using the endopterygota_odb10 reference set. As these are different databases with different gene counts, the author should clearly distinguish between the two analyses.
- There are numerical inconsistencies for genome size estimation using GenomeScope2. Figure 2 reports the genome length as 261,442,079 bp (equivalent to 261.44 Mb), whereas the text reports 261.67 Mb. This should be corrected to 261.44 Mb. Similarly, the repeat content reported in Figure 2 is 32.1%, but in the text it is 32.08%.

- The total scaffold count is inconsistently reported. Table 2 states 523 scaffolds for haplotype 1, whereas the snail plot in Figure 5 displays a total scaffold count of 524. The author should verify this. 

Are sufficient details of methods and materials provided to allow replication by others?

Yes

Is the rationale for creating the dataset(s) clearly described?

Yes

Are the datasets clearly presented in a useable and accessible format?

Yes

Are the protocols appropriate and is the work technically sound?

Yes

Reviewer Expertise:

Genome evolution

We confirm that we have read this submission and believe that we have an appropriate level of expertise to confirm that it is of an acceptable scientific standard.

# Reviewer report: Reviewer response for version 1

**DOI:** 10.21956/wellcomeopenres.28758.r151706

**Published:** 2026-04-06

**Authors:** Stuart J.E. Baird

**Affiliations:** 1 Academy of Sciences of the Czech Republic, Národní, Staré Město, Czech Republic

The report largely follows the (excellent) standard DToL template. I find two minor issues:

 (1) The rationale for the work is stated in the abstract "This assembly was generated as part of the Darwin Tree of Life project, which produces reference genomes for eukaryotic species found in Britain and Ireland." However, the genome report concerns a species collected in Finland and not reported in the UK "Only *S. fenestralis* and *S. niger* have been reported from the UK ( Stubbs & Drake, 2014)." (Background). The report could expand on the fact that "The reference genome assembly of *Scenopinus jerei* provides the first genomic resource for Scenopinidae." (Background) ...meaning the report is an important landmark for a family and genus found in Britain and Ireland.

 (2) Figure 3. Hi-C contact map of the *Scenopinus jerei* genome assembly: In contrast to standard DToL report contact maps, an unnecessary empty (grey) border has been left at bottom and right. This does no harm, but is disturbing, giving the reader the impression something is missing, and reduces the resolution of the data-full region.

Are sufficient details of methods and materials provided to allow replication by others?

Yes

Is the rationale for creating the dataset(s) clearly described?

No

Are the datasets clearly presented in a useable and accessible format?

Yes

Are the protocols appropriate and is the work technically sound?

Yes

Reviewer Expertise:

Admixture genomics

I confirm that I have read this submission and believe that I have an appropriate level of expertise to confirm that it is of an acceptable scientific standard.

# Reviewer report: Reviewer response for version 1

**DOI:** 10.21956/wellcomeopenres.28758.r151051

**Published:** 2026-04-03

**Authors:** Matsapume Detcharoen

**Affiliations:** 1 Prince of Songkla University, Hat Yai, Thailand

This manuscript describes the reference genome assembly for the window fly Scenopinus jerei. The authors note that this species was only formally described in 2021, and that this assembly provides the first genomic resource for the family Scenopinidae.

 Technically, the manuscript reports sequencing and assembly of the genome from a male specimen collected in Finland, with additional RNA sequencing from another specimen. The main result is a genome assembly with two haplotypes, including the X and Y chromosomes, and mitochondrial genome was also assembled. The assembly is reported as high quality.

 The main strengths are its novelty and assembly quality. This is the first genome resource for the fly family Scenopinidae, so it fills a real gap in biodiversity genomics. The species itself was only described recently, which makes the dataset especially useful for future work on window fly evolution.

Are sufficient details of methods and materials provided to allow replication by others?

Yes

Is the rationale for creating the dataset(s) clearly described?

Yes

Are the datasets clearly presented in a useable and accessible format?

Yes

Are the protocols appropriate and is the work technically sound?

Yes

Reviewer Expertise:

Molecular ecology

I confirm that I have read this submission and believe that I have an appropriate level of expertise to confirm that it is of an acceptable scientific standard.
